# Long-Term Surveillance After Curative Endoscopic Submucosal Dissection for Early Gastric Cancer: Distinct Clinicopathologic Characteristics of Early and Late Metachronous Neoplasia

**DOI:** 10.3390/jcm15218435

**Published:** 2026-10-29

**Authors:** Moon Kyung Joo, Seung Han Kim, Won Shik Kim, Beom Jae Lee

**Affiliations:** Division of Gastroenterology, Department of Internal Medicine, Korea University Guro Hospital, Korea University College of Medicine, 148, Gurodong-ro, Guro-gu, Seoul 08308, Republic of Korea; kimseunghan09@gmail.com (S.H.K.); ws907568@gmail.com (W.S.K.); l85210@korea.ac.kr (B.J.L.)

**Keywords:** stomach neoplasia, endoscopy, gastrointestinal, surveillance

## Abstract

**Background**: Endoscopic submucosal dissection (ESD) is an established treatment for early gastric cancer (EGC) meeting curative resection criteria. We examined clinicopathologic characteristics associated with early and late metachronous neoplasia after curative ESD. **Methods**: We retrospectively analyzed 185 patients who developed metachronous neoplasia among 1200 patients who underwent curative ESD for EGC. Metachronous neoplasia detected within 60 months was classified as early, and late if it is detected after 60 months. **Results**: Of the 185 patients with metachronous neoplasia (15.4%), 116 (62.7%) had early and 69 (37.3%) had late metachronous neoplasia; 102 developed cancer and 83 developed adenoma only. Undifferentiated histology of the index lesion was associated with late metachronous neoplasia overall (adjusted odds ratio [OR], 10.73; 95% confidence interval [CI], 1.27–90.35; *p* = 0.029). In the adenoma subgroup, undifferentiated histology occurred exclusively in patients with late metachronous adenoma (11.1% vs. 0%; *p* = 0.032), but its adjusted effect could not be estimated because of complete separation. Younger age was associated with late metachronous adenoma (adjusted OR per year, 0.937; 95% CI, 0.881–0.997; *p* = 0.040). In the cancer subgroup, serum triglyceride was associated with late metachronous cancer in univariable analysis (OR per 10 mg/dL, 1.064; 95% CI, 1.004–1.128; *p* = 0.036), but not after multivariable adjustment (*p* = 0.101). **Conclusions**: More than one-third of metachronous neoplasia were detected beyond five years after curative ESD. Undifferentiated histology was associated with late metachronous neoplasia overall and in the adenoma subgroup, although the latter association could not be adjusted. These findings warrant validation in larger cohorts.

## 1. Introduction

Endoscopic submucosal dissection (ESD) is an established treatment for early gastric cancer (EGC) that fulfills established criteria for curative endoscopic resection because it enables en bloc resection while preserving the stomach and can provide favorable oncologic outcomes in appropriately selected patients [1]. Organ preservation following ESD is associated with lower procedural morbidity, better postoperative quality of life, and maintenance of gastric function, supporting its use in appropriately selected patients with EGC. Nevertheless, preservation of the stomach also leaves the background gastric mucosa susceptible to subsequent neoplastic transformation, creating an ongoing risk of metachronous recurrence during long-term follow-up [2]. This unique characteristic distinguishes endoscopic treatment from gastrectomy and makes surveillance an integral component of post-ESD management.

Metachronous gastric neoplasia develop in approximately 10–20% of patients during follow-up after curative ESD despite complete removal of the initial lesion [2,3,4,5,6]. Persistent mucosal alterations, including chronic inflammation, atrophic gastritis, intestinal metaplasia, and field cancerization, are believed to contribute to continued carcinogenesis within the remnant stomach [7,8]. Consequently, current clinical practice guidelines recommend regular endoscopic surveillance after curative ESD [9]. However, surveillance protocols are often standardized and do not adequately account for differences in individual recurrence risk or the timing of recurrence. Previous studies have mainly focused on identifying patients at increased risk of metachronous recurrence. Advanced age, persistent *Helicobacter pylori* (*H. pylori*) infection, severe gastric atrophy, synchronous neoplasia, and several inflammatory or metabolic factors have all been associated with recurrence after ESD [10].

Although these studies have improved understanding of who develops metachronous neoplasia, less attention has been paid to the timing of recurrence. In particular, relatively few investigations have examined whether patients developing recurrence within the first 5 years differ biologically or clinically from those experiencing recurrence after prolonged disease-free intervals. This distinction may have important clinical implications. Early and late metachronous recurrence may not represent the same biological process. Early recurrence may be more closely related to pre-existing mucosal carcinogenic changes present at the time of index treatment [8], whereas late recurrence may reflect ongoing carcinogenesis associated with long-term host factors, metabolic abnormalities, or persistent molecular alterations within the gastric mucosa [11]. Identifying factors specifically associated with late recurrence could therefore improve long-term risk stratification and help determine which patients may benefit from surveillance beyond the conventional 5-year period.

In this study, we compared the clinicopathologic characteristics of patients with early and late metachronous neoplasia after curative ESD for EGC. We focused on timing of metachronous neoplasia, rather than on the risk of recurrence in the entire post-ESD population. We hypothesized that early and late metachronous neoplasia may be associated with different clinicopathologic profiles.

Highlights

More than one-third of metachronous neoplasia were detected more than five years after curative ESD for early gastric cancer. Undifferentiated histology was associated with late metachronous neoplasia overall and occurred exclusively in the late metachronous adenoma subgroup; younger age was associated with late metachronous adenoma.

## 2. Materials and Methods

### 2.1. Study Design and Patients

We performed a retrospective study of consecutive patients who underwent curative ESD for EGC at a tertiary referral hospital between January 2006 and December 2023. During the study period, 1200 patients achieved curative resection and completed at least 12 months of surveillance. Among them, 185 patients subsequently developed metachronous gastric neoplasia and constituted the study population. Because the primary aim of this study was to investigate clinicopathologic characteristics associated with the timing of metachronous neoplasia rather than to identify risk factors for recurrence itself, only patients who developed metachronous neoplasia were included in the comparative analyses.

### 2.2. ESD Procedure and Follow-Up

All ESD procedures were performed by experienced endoscopists using standard ESD techniques in accordance with established procedural principles [12,13]. Lesion margins were delineated using high-definition white-light endoscopy with image-enhanced endoscopy when appropriate. After submucosal injection, circumferential mucosal incision and submucosal dissection were performed to achieve en bloc resection. Following curative ESD, surveillance endoscopy was routinely scheduled at 3 and 6 months and subsequently at 12-month intervals. Abdominopelvic computed tomography was performed according to institutional surveillance protocols and individual clinical indications. Histopathological examination was performed whenever suspicious lesions were identified during follow-up. *H. pylori* infection was assessed using a rapid urease test or histological examination of gastric biopsy specimens with special staining. We prescribed standard triple or concomitant therapy regimen as a first-line therapy, whereas bismuth-based quadruple therapy was used as second-line treatment. Successful eradication was confirmed by a urea breath test or histological examination with special staining.

### 2.3. Data Collection

Demographic, clinical, laboratory, endoscopic, and pathological data were retrieved from a prospectively maintained institutional ESD database and electronic medical records. Baseline demographic variables included age, sex, body mass index (BMI), smoking history, family history of gastric cancer, underlying comorbidities, and the use of antithrombotic medications. Laboratory parameters obtained before ESD included complete blood cell counts, erythrocyte sedimentation rate (ESR), serum albumin, fasting glucose, lipid profiles, serum creatinine, and C-reactive protein (CRP). In addition, inflammatory and nutritional indices, including the neutrophil-to-lymphocyte ratio (NLR), platelet-to-lymphocyte ratio (PLR), prognostic nutritional index (PNI), and triglyceride–glucose (TyG) index, were calculated using established methods. Clinicopathologic characteristics of the index lesion included tumor location, macroscopic appearance, ulcerative findings, tumor size, degree of endoscopic atrophy, histological differentiation, Lauren classification, depth of invasion, indication criteria for ESD, *H. pylori* infection status, synchronous gastric neoplasia, and the occurrence of multiple metachronous lesions.

### 2.4. Definitions

Curative resection was determined according to the Korean clinical practice guidelines for endoscopic resection of early gastrointestinal cancer, based on en bloc resection, histological differentiation, tumor size, depth of invasion, ulcerative findings, resection margin status, lymphovascular invasion, and the absolute or expanded indication criteria for endoscopic resection. Metachronous neoplasia was defined as a newly developed gastric adenoma or adenocarcinoma detected at a site distinct from the index lesion at least 12 months after curative ESD [14]. Early metachronous neoplasia was defined as an adenoma or adenocarcinoma detected within 60 months after ESD, whereas late metachronous neoplasia was defined as a lesion detected beyond 60 months. Additional subgroup analyses were performed separately for patients with metachronous adenoma and metachronous cancer. A 60-month cutoff was selected a priori to distinguish early from late metachronous neoplasia because a 5-year disease-free interval is generally regarded as a clinically meaningful milestone in oncologic follow-up [15]. Although this threshold does not necessarily represent a biological transition, it provides a practical framework for evaluating whether recurrence timing is associated with distinct clinicopathologic characteristics. This classification was intended to differentiate recurrences that may reflect pre-existing carcinogenic alterations from those potentially arising through ongoing carcinogenesis during prolonged surveillance.

Patients were classified as having early or late metachronous neoplasia in the overall cohort according to the timing of their first metachronous neoplasia. For the cancer subgroup, classification was based on the timing of the first metachronous carcinoma, whereas the adenoma-only subgroup included patients who developed metachronous adenoma without subsequent carcinoma during follow-up. Thus, one patient who developed adenoma within 60 months and subsequently developed carcinoma after 60 months was classified as having early group in the overall cohort and late group in the cancer subgroup.

Endoscopic atrophy was graded according to the Kimura–Takemoto classification and categorized as C1–2, C3–O1, or O2–3 [16]. Persistent *H. pylori* infection was defined as documented infection that remained untreated or persisted despite eradication therapy during follow-up. The prognostic nutritional index was calculated as 10 × serum albumin (g/dL) + 0.005 × peripheral lymphocyte count (/mm^3^) [17]. The triglyceride–glucose (TyG) index was calculated as ln [fasting triglyceride (mg/dL) × fasting glucose (mg/dL)/2] [18].

### 2.5. Statistical Analysis

Continuous variables were assessed according to their distributions and are presented as mean ± standard deviation for approximately normally distributed variables or median [interquartile range] for skewed variables. Categorical variables are expressed as numbers and percentages. Approximately normally distributed continuous variables were compared using Welch’s *t*-test, whereas skewed continuous variables were compared using the Mann–Whitney U test. Categorical variables were analyzed using the chi-square test or Fisher’s exact test, as appropriate. Logistic regression analysis was performed to examine factors associated with late rather than early metachronous neoplasia. To preserve statistical information and avoid information loss, continuous predictors were retained as continuous variables rather than dichotomized by data-derived cutoffs. Variables showing potential associations in univariable analyses and clinically relevant variables were considered for multivariable models, with the number of covariates restricted according to subgroup sample size. Multicollinearity was assessed using variance inflation factors (VIFs), and model complexity was evaluated by calculating the number of late events per predictor. To address potential sparse-data bias and overfitting, Firth penalized logistic regression was performed as a sensitivity analysis. In the cancer subgroup, an additional reduced model including age, triglycerides, and undifferentiated histology was evaluated. Internal validation of this reduced model was performed using 500 bootstrap resamples to estimate optimism-corrected discrimination. Odds ratios (ORs) and 95% confidence intervals (CIs) were calculated. For clinical interpretability, ORs for white blood cell count and serum triglyceride were expressed per 1000/µL and per 10 mg/dL increase, respectively. All statistical tests were two-sided, and a *p* value < 0.05 was considered statistically significant.

### 2.6. Ethics Statement

The study protocol was approved by the Institutional Review Board of Korea University Guro Hospital (IRB No. 2023GR0458), which waived the requirement for informed consent because of the retrospective nature of the study. The study was conducted in accordance with the ethical principles of the Declaration of Helsinki.

## 3. Results

### 3.1. Patient Characteristics

During the study period, 1200 patients underwent curative ESD for EGC and completed at least 12 months of surveillance. Their median follow-up duration was 59.2 months (interquartile range, 33.5–81.5 months), and 558 patients (46.5%) were followed for more than five years. Among them, 185 patients (15.4%) developed metachronous gastric neoplasia and were included in the analysis. Of these, 116 (62.7%) experienced metachronous neoplasia within 60 months (early metachronous neoplasia), and 69 (37.3%) developed after 60 months (late metachronous neoplasia). Overall, 83 patients (44.9%) developed adenoma only and 102 (55.1%) developed cancer. Among patients with metachronous adenoma only, 56 (67.4%) had early and 27 (32.6%) had late. Among those with metachronous cancer, 59 (57.8%) had early and 43 (42.2%) had late. The difference in the distribution of early and late metachronous neoplasia between the overall cohort and the histological subgroups was attributable to one patient who developed early metachronous adenoma followed by late metachronous cancer.

### 3.2. Baseline Clinical Characteristics According to Timing of Metachronous Neoplasia

Baseline clinical characteristics of the early and late groups are summarized in Table 1. Patients with late metachronous neoplasia were significantly younger than those with early metachronous neoplasia (63.9 ± 8.3 vs. 66.9 ± 7.9 years, *p* = 0.018). No significant differences were observed with respect to sex, smoking status, family history of gastric cancer, BMI, or underlying comorbidities. Among laboratory parameters, serum albumin was significantly higher in the late group (4.26 ± 0.23 vs. 4.17 ± 0.27 g/dL, *p* = 0.023), as was the PNI (54.0 ± 5.6 vs. 52.1 ± 4.4, *p* = 0.020). No significant between-group differences were observed in inflammatory markers, including WBC count, NLR, PLR, ESR, and CRP. Serum lipid profiles, fasting glucose, and the TyG index were also comparable between the groups.

### 3.3. Clinicopathologic Characteristics of Early and Late Metachronous Neoplasia

Clinicopathologic characteristics of the index lesions are presented in Table 2. Tumor location, macroscopic morphology, ulcerative findings, tumor size, degree of endoscopic atrophy, depth of invasion, ESD indication criteria, *H. pylori* infection status, synchronous neoplasia, and multiple metachronous neoplasia did not differ significantly between the groups. In contrast, histological characteristics differed according to timing of metachronous neoplasia. Undifferentiated-type histology was significantly more frequent in patients with late metachronous neoplasia than in those with early metachronous neoplasia (10.1% vs. 0.9%, *p* = 0.005). Lauren classification also differed significantly between groups (*p* = 0.025), with diffuse or mixed histology occurring more frequently in the late group. Post-ESD histologic diagnosis differed between the groups (*p* = 0.025), with poorly differentiated adenocarcinoma and signet-ring cell carcinoma more frequent in the late group.

### 3.4. Factors Associated with Late Metachronous Neoplasia

In univariable logistic regression analysis using continuous predictors (Table 3), younger age (OR per year, 0.955; 95% CI, 0.920–0.992; *p* = 0.018), higher serum albumin (OR per 1 g/dL, 3.866; 95% CI, 1.135–13.173; *p* = 0.031), higher PNI (OR per unit, 1.081; 95% CI, 1.015–1.152; *p* = 0.016), and undifferentiated histology (OR, 12.984; 95% CI, 1.562–107.950; *p* = 0.018) were associated with late metachronous metachronus neoplasia. After multivariable adjustment, undifferentiated histology remained the only factor that remained statistically significant in the adjusted model (OR, 10.727; 95% CI, 1.274–90.352; *p* = 0.029). Age, serum albumin, and PNI were no longer statistically significant in the adjusted model. In a sensitivity analysis using Firth penalized logistic regression, the association persisted (adjusted OR, 7.511; 95% CI, 1.569–72.765; *p* = 0.010); however, the wide CI indicated substantial uncertainty in the effect estimate (Supplementary Table S1). In sensitivity analyses using alternative thresholds of 48 and 72 months, undifferentiated histology remained associated with later metachronous neoplasia at 48 months (adjusted OR, 5.247; 95% CI, 1.106–50.609; *p* = 0.036), whereas the association did not reach statistical significance at 72 months (adjusted OR, 3.184; 95% CI, 0.805–14.289; *p* = 0.098) (Supplementary Table S2).

In summary undifferentiated histology of the index lesion showed an association with late rather than early metachronous neoplasia among patients who developed metachronous lesions, although this finding should be interpreted cautiously because of the small number of undifferentiated cases and the wide range of CI.

### 3.5. Subgroup Analysis According to Histology

#### 3.5.1. Late Metachronous Cancer

Clinical and clinicopathologic characteristics of patients with metachronous cancer are summarized in Table 4, Table 5 and Table 6. Among the 102 patients with recurrent cancer, 59 (57.8%) developed metachronous cancer within 60 months, whereas 43 (42.2%) experienced beyond 60 months after curative ESD. Late metachronous cancer showed numerically lower age (64.9 ± 8.1 vs. 67.6 ± 8.0 years, *p* = 0.097), higher BMI (24.9 ± 2.9 vs. 23.9 ± 2.6 kg/m^2^, *p* = 0.058), and higher PNI (54.4 ± 6.0 vs. 52.4 ± 4.4, *p* = 0.069). Serum triglyceride was higher numerically in the late group (median 146.5 [84.2–212] vs. 118 [83.5–154] mg/dL), but the between-group difference was not statistically significant (*p* = 0.097). Other laboratory and clinicopathologic characteristics were also comparable between groups; undifferentiated histology was more frequent in the late group (9.3% vs. 1.7%) but did not reach statistical significance (*p* = 0.159).

In continuous-variable logistic regression analysis (Table 6), serum triglyceride was associated with late metachronous cancer in univariable analysis (OR per 10 mg/dL increase, 1.064; 95% CI, 1.004–1.128; *p* = 0.036). Age, BMI, WBC count, PNI, and undifferentiated histology did not reach conventional statistical significance, although several showed borderline associations. After multivariable adjustment, however, serum triglyceride was no longer statistically significant (adjusted OR per 10 mg/dL, 1.052; 95% CI, 0.990–1.117; *p* = 0.101), and no variable remained independently associated with late metachronous cancer in this subgroup.

No substantial multicollinearity was observed in the multivariable models (maximum VIF, 1.95 in the overall cohort and 1.87 in the cancer subgroup). The number of late events per predictor was 17.3 in the overall model and 6.3 in the original cancer subgroup model. In a reduced three-variable Firth logistic regression model for the cancer subgroup, triglyceride levels were associated with late metachronous cancer (adjusted OR per 10 mg/dL, 1.052; 95% CI, 1.002–1.120; *p* = 0.039), whereas undifferentiated histology was not statistically significant (adjusted OR, 3.334; 95% CI, 0.506–36.121; *p* = 0.213) (Supplementary Table S3). However, the association between triglyceride levels and late metachronous cancer was not statistically significant in the original multivariable model. Therefore, this model-dependent finding should be interpreted cautiously. Bootstrap internal validation yielded an apparent AUC of 0.646 and an optimism-corrected AUC of 0.614, indicating limited discriminatory performance (Supplementary Table S4).

#### 3.5.2. Late Metachronous Adenoma

Clinical and clinicopathologic characteristics of patients with metachronous adenoma only are summarized in Table 7, Table 8 and Table 9. Of the 83 patients in this subgroup, 56 (67.4%) had early metachronous adenoma and 27 (32.6%) had late metachronous adenoma. Patients with late metachronous adenoma were numerically younger than those with early metachronous adenoma (62.3 ± 8.4 vs. 66.1 ± 7.9 years; *p* = 0.055), and their CRP levels were numerically higher (0.88 [0.55–1.63] vs. 0.53 [0.27–1.43] mg/dL; *p* = 0.077). Histological differentiation and Lauren classification differed significantly between groups. Notably, undifferentiated histology of the index lesion occurred exclusively in the late group (3/27 [11.1%] vs. 0/56 [0%]; *p* = 0.032). Because the early group had no undifferentiated cases, conventional logistic regression could not reliably estimate its OR; therefore, this variable was excluded from the conventional multivariable model. Although Firth penalized regression yielded an estimate (adjusted OR, 12.704; 95% CI,1.123–1749.115; *p* = 0.039), the extremely wide CI indicated substantial uncertainty, and this finding was considered exploratory and was not interpreted as evidence of an independent association (Supplementary Table S1). In the resulting model (Table 9), younger age was associated with late metachronous adenoma (adjusted OR per year, 0.937; 95% CI, 0.881–0.997; *p* = 0.040), whereas CRP was not statistically significant (adjusted OR per 1 mg/dL, 1.370; 95% CI, 0.974–1.928; *p* = 0.071).

## 4. Discussion

In this retrospective study, we compared the clinicopathologic characteristics of patients with early and late metachronous neoplasia after curative ESD for EGC. Three findings merit attention. First, 37.3% of metachronous neoplasia were detected more than five years after ESD. Second, undifferentiated histology of the index lesion was associated with late metachronous neoplasia in the overall analysis. Third, the subgroup analyses differed: in the adenoma subgroup, undifferentiated histology occurred exclusively among patients with late metachronous adenoma and showed a significant unadjusted association, although its adjusted effect could not be estimated; younger age remained associated with late metachronous adenoma after adjustment. In the cancer subgroup, serum triglyceride was associated with late metachronous cancer in univariable analysis but not after multivariable adjustment. Our findings suggest that certain clinicopathological characteristics may be associated with the timing of metachronous neoplasia after curative ESD. However, because the analysis was restricted to patients who developed metachronous lesions, these associations cannot be used to estimate the absolute risk of late metachronous neoplasia in the overall ESD population. Furthermore, these findings should be considered exploratory rather than evidence of distinct biological mechanisms or established predictors of late recurrence.

A notable finding was that 37.3% of metachronous neoplasia in this metachronous neoplasia-only cohort were detected more than five years after curative ESD. Although periodic endoscopic surveillance after curative ESD is widely recommended, its optimal duration remains uncertain. Our findings demonstrate that delayed metachronous neoplasia occur during prolonged follow-up. However, because the analysis was restricted to patients who developed metachronous neoplasia, the proportion observed here does not estimate the cumulative incidence of late recurrence in the entire post-ESD population or establish an optimal surveillance interval.

Several mechanisms may explain the occurrence of late metachronous recurrence. Unlike gastrectomy, ESD preserves the stomach and therefore does not eliminate the carcinogenic background mucosa. Even after complete removal of the index lesion, chronic inflammation, extensive gastric atrophy, intestinal metaplasia, and the underlying field defect of the gastric mucosa may persist, providing a biological basis for independent neoplastic transformation during long-term follow-up [19,20]. This concept is supported by accumulating evidence demonstrating that molecular and epigenetic alterations, particularly aberrant DNA methylation, may persist in the gastric mucosa even after successful *H. pylori* eradication, thereby maintaining a field defect that predisposes patients to subsequent gastric neoplasia [21]. Accordingly, late recurrence may reflect ongoing gastric carcinogenesis, although delayed detection of pre-existing lesions cannot be excluded during long-term follow-up. However, our findings do not establish distinct underlying biological mechanisms. These associations should therefore be considered hypothesis-generating and require further validation in larger studies incorporating molecular and histopathological assessments.

One of the most notable findings was the association between undifferentiated histology of the index lesion and late metachronous neoplasia. Patients with late metachronous neoplasia exhibited a substantially higher frequency of undifferentiated histology than those with early metachronous neoplasia (10.1% vs. 0.9%), and this association remained significant in multivariable analysis (adjusted OR, 10.727; 95% CI, 1.274–90.352). Although undifferentiated-type EGC has traditionally been associated with lymph node metastasis and more aggressive biological behavior, its relationship with the timing of metachronous neoplasia has received relatively little attention. One possible explanation is that undifferentiated histology reflects a distinct molecular phenotype and associated carcinogenic processes even after curative treatment [22]. Alternatively, undifferentiated histology may serve as a surrogate marker for a broader field defect within the gastric mucosa, reflecting susceptibility to multicentric gastric carcinogenesis [23]. The wide confidence interval in our analysis, however, reflects the small number of undifferentiated lesions and warrants cautious interpretation and external validation. Although the association between undifferentiated histology and late metachronous neoplasia persisted in Firth penalized logistic regression, the small number of undifferentiated cases and wide CIs warrant cautious interpretation. Therefore, this finding should be considered exploratory rather than evidence of an established independent predictor. Furthermore, sensitivity analyses using alternative cutoffs showed a consistent direction of association for undifferentiated histology; however, statistical significance was not maintained at the 72-month threshold. This finding suggests that the observed association may be sensitive to the selected cutoff and should be interpreted cautiously.

Younger age was also associated with later metachronous adenoma, although the pattern differed by analysis. In the overall cohort, patients with late metachronous neoplasia were younger, but age did not remain significant after multivariable adjustment. In the adenoma subgroup, however, younger age was associated with late metachronous adenoma (adjusted OR per year, 0.937; *p* = 0.040). At first glance, this appears inconsistent with previous studies identifying advanced age as a risk factor for the occurrence of metachronous gastric neoplasia after curative ESD [24,25]. The clinical question in the present study is different: rather than comparing patients with and without recurrence, we compared patients who ultimately recurred according to whether metachronous neoplasia occurred before or after five years. Differences in follow-up duration and surveillance opportunities may partly contribute to the observed association. Accordingly, the present findings should be interpreted as factors associated with timing among patients who experienced metachronous neoplasia, rather than as evidence that younger age increases the overall risk of metachronous neoplasia.

Serum triglyceride showed an exploratory association with late metachronous cancer. Median triglyceride levels were numerically higher in the late group, but the between-group comparison was not statistically significant. When triglyceride was analyzed as a continuous predictor, it was associated with late metachronous cancer in univariable logistic regression, but the association was attenuated after adjustment. Thus, our data do not establish triglyceride as an independent marker of late metachronous cancer. Altered lipid metabolism has been implicated in carcinogenesis [26,27], but the relationship observed here requires confirmation in larger studies before it can inform clinical risk stratification.

The adenoma and cancer subgroup analyses suggest potentially different associations with timing of metachronous neoplasia, although the findings should be interpreted cautiously. In the adenoma subgroup, undifferentiated histology of the index lesion occurred exclusively among patients with late metachronous adenoma. This significant between-group association is noteworthy, but the absence of undifferentiated cases in the early group produced complete separation, preventing reliable estimation of an adjusted OR using conventional logistic regression. The inability to estimate an adjusted effect does not imply the absence of an association. Younger age remained associated with late adenoma after adjustment in a model excluding undifferentiated histology. In the cancer subgroup, no variable remained statistically significant after multivariable adjustment. Although the overall and adenoma findings for undifferentiated histology are directionally consistent, the adenoma result was based on only three cases, and the subgroup analyses were exploratory. Larger multicenter studies are needed to validate these observations.

These findings have implications for future research on post-ESD surveillance. The detection of metachronous neoplasia beyond five years underscores the importance of considering prolonged follow-up in clinical practice. Undifferentiated histology of the index lesion may provide information about timing among patients who develop metachronous neoplasms. However, this metachronous neoplasia-only analysis cannot estimate absolute risk of late metachronous neoplasia or determine which patients should receive intensified surveillance. Prospective studies of the entire post-ESD population are needed to develop and validate risk-adapted surveillance strategies. Artificial intelligence (AI) may offer additional opportunities to improve long-term endoscopic surveillance after ESD. Previous colorectal studies have demonstrated the potential of AI-assisted lesion characterization and data-driven risk stratification to support surveillance strategies [28,29]. Similar approaches integrating endoscopic findings and clinicopathological characteristics may eventually facilitate individualized surveillance for metachronous gastric neoplasia.

This study has several limitations. First, its retrospective single-center design introduces the possibility of selection bias and residual confounding. Second, only patients who developed metachronous neoplasia were included in the comparative analyses because the primary objective was to evaluate differences according to the timing rather than predictors of recurrence itself. Consequently, the identified factors should be interpreted as characteristics associated with late versus early metachronous neoplasia among patients who experienced recurrence, not as predictors applicable to the entire ESD population. Third, the late subgroups were small, particularly for undifferentiated histology, resulting in wide confidence intervals and, in the adenoma subgroup, complete separation that prevented estimation of the undifferentiated-histology OR. Furthermore, variations in follow-up duration, with fewer than half of the entire cohort followed for more than five years, may have influenced the detection of late metachronous neoplasia. Fourth, the multivariable subgroup models may remain vulnerable to overfitting and should be considered exploratory. Although reduced Firth regression and bootstrap internal validation were performed to address potential overfitting, the limited number of late metachronous cases, particularly in the subgroup analyses, remains an important limitation. Fifth, molecular biomarkers, genetic alterations, and detailed assessments of the background gastric mucosa, including intestinal metaplasia staging, were unavailable. Furthermore, several potentially important confounding factors, including the severity of intestinal metaplasia, autoimmune gastritis, *H. pylori* eradication timing, and molecular alterations, were not comprehensively assessed. Therefore, residual confounding cannot be excluded, and the proposed biological mechanisms underlying differences in timing of metachronous neoplasia remain exploratory. Finally, the observational design does not establish causal relationships between the identified characteristics and delayed carcinogenesis.

This study also has strengths. By comparing early and late metachronous neoplasia among patients who developed metachronous neoplasia, we examined an aspect of post-ESD outcomes that has received less attention than metachronous neoplasia itself. The available follow-up enabled us to characterize neoplasia detected beyond five years, although our findings require external validation. Although our findings may provide insights into the potential individualization of long-term endoscopic surveillance after curative ESD, further validation is required before these associations can be used to guide surveillance intervals in clinical practice.

## 5. Conclusions

More than one-third of metachronous neoplasia in this metachronous neoplasia-only cohort were detected more than five years after curative ESD for EGC. Undifferentiated histology of the index lesion was associated with late metachronous neoplasia in the overall analysis. In the adenoma subgroup, undifferentiated histology occurred exclusively among late metachronous adenomas and showed a significant unadjusted association, but its adjusted effect could not be estimated because of complete separation; younger age remained associated with late metachronous adenoma after adjustment. In the cancer subgroup, the univariable association with triglyceride was attenuated after multivariable adjustment. These exploratory findings warrant validation in larger multicenter cohorts and should not be interpreted as evidence of distinct biological mechanisms or established predictors of late metachronous neoplasia.

## Figures and Tables

**Table 1:** Baseline Characteristics of Early and Late Metachronous Neoplasia.

Variable	Early (n = 116)	Late (n = 69)	*p*-Value
Age (years)	66.9 ± 7.9	63.9 ± 8.3	0.018
Gender			0.734
Male	90 (77.6%)	55 (79.7%)	
Female	26 (22.4%)	14 (20.3%)	
Family history of GC	12 (10.3%)	6 (8.7%)	0.714
Smoking			0.977
Never smoker	67 (57.8%)	40 (58.0%)	
Past or current smoker	49 (42.2%)	29 (42.0%)	
Hypertension	62 (53.4%)	30 (43.5%)	0.190
Diabetes mellitus	33 (28.4%)	16 (23.2%)	0.433
Heart disease	11 (9.5%)	7 (10.1%)	0.883
Cerebrovascular accident	7 (6.0%)	2 (2.9%)	0.488
End-stage renal disease	4 (3.4%)	0 (0.0%)	0.299
Aspirin	12 (10.3%)	7 (10.1%)	0.965
Other antithrombotic agent	19 (16.4%)	6 (8.7%)	0.139
Height (m)	1.6 ± 0.1	1.6 ± 0.1	0.926
Weight (kg)	65.5 ± 10.1	65.8 ± 9.6	0.831
BMI (kg/m^2^)	24.4 ± 3.2	24.6 ± 2.9	0.764
WBC count (/µL)	6300 [5450–7550]	6700 [5800–7900]	0.113
Neutrophil count (/µL)	3522 [2925.5–4435]	3781 [3289–4755]	0.132
Lymphocyte count (/µL)	2041.2 [1703.8–2380.7]	2129.9 [1593–2748.2]	0.217
Platelet count (×10^3^/µL)	219.8 ± 57.2	214.7 ± 53.9	0.544
NLR	1.81 [1.34–2.32]	1.81 [1.29–2.51]	0.769
PLR	109.65 [83.84–128.04]	95.09 [72.46–127.31]	0.139
ESR (mm/hr)	13 [6–23]	11 [7–22]	0.891
Albumin (g/dL)	4.17 ± 0.27	4.26 ± 0.23	0.023
PNI	52.1 ± 4.4	54.0 ± 5.6	0.020
Total cholesterol (mg/dL)	171.1 ± 35.4	173.8 ± 37.3	0.634
HDL (mg/dL)	48.8 ± 12.7	48.3 ± 13.9	0.822
LDL (mg/dL)	98.5 ± 32.7	98.9 ± 36.3	0.942
TG (mg/dL)	124 [87–167]	136 [86.5–203]	0.314
Fasting glucose (mg/dL)	105 [96–129]	106 [94–123]	0.401
TyG index	8.84 ± 0.59	8.89 ± 0.75	0.662
Serum creatinine (mg/dL)	0.83 [0.70–0.94]	0.81 [0.72–0.88]	0.311
CRP (mg/dL)	0.61 [0.33–1.56]	0.88 [0.48–1.73]	0.166

Note: Data are presented as n (%), mean ± SD, or median [IQR], as appropriate. SD, standard deviation; GC, gastric cancer; BMI, body mass index; WBC, white blood cell; IQR, interquartile range; NLR, neutrophil-to-lymphocyte ratio; PLR, platelet-to-lymphocyte ratio; ESR, erythrocyte sedimentation rate; PNI, prognostic nutritional index; HDL, high-density lipoprotein; LDL, low-density lipoprotein; TG, triglyceride; TyG, triglyceride–glucose index; CRP, C-reactive protein.

**Table 2:** Clinicopathologic Characteristics of Early and Late Metachronous Neoplasia.

Variable	Early (n = 116)	Late (n = 69)	*p*-Value
Lesion location			0.738
Lower third	71 (61.2%)	46 (66.7%)	
Mid third	40 (34.5%)	20 (29.0%)	
Upper third	5 (4.3%)	3 (4.3%)	
Lesion shape			0.423
Elevated (I, IIa)	30 (25.9%)	22 (31.9%)	
Flat (IIb)	41 (35.3%)	27 (39.1%)	
Depressed (IIc)	44 (37.9%)	20 (29.0%)	
Presence of ulcer	13 (11.2%)	6 (8.7%)	0.586
Tumor size (mm)	14.5 [8–23.2]	16 [8–23]	0.917
Atrophy			0.986
C1–2	3 (2.6%)	2 (2.9%)	
C3–O1	38 (32.8%)	22 (31.9%)	
O2–3	75 (64.7%)	45 (65.2%)	
Post-ESD diagnosis			0.025
WD adenocarcinoma	68 (58.6%)	36 (52.2%)	
MD adenocarcinoma	47 (40.5%)	26 (37.7%)	
PD adenocarcinoma	1 (0.9%)	4 (5.8%)	
SRC	0 (0.0%)	3 (4.3%)	
Differentiation			0.005
Differentiated type	115 (99.1%)	62 (89.9%)	
Undifferentiated type	1 (0.9%)	7 (10.1%)	
Lauren classification			0.025
Intestinal	115 (99.1%)	63 (91.3%)	
Diffuse	1 (0.9%)	5 (7.2%)	
Mixed	0 (0.0%)	1 (1.4%)	
Depth of invasion			1.000
Mucosa	109 (94.0%)	65 (94.2%)	
Submucosa	7 (6.0%)	4 (5.8%)	
Criteria			0.321
Absolute	57 (49.6%)	29 (42.0%)	
Expanded	58 (50.4%)	40 (58.0%)	
*H. pylori* infection			0.627
Negative or eradicated	103 (92.0%)	62 (89.9%)	
Persistent	9 (8.0%)	7 (10.1%)	
Unevaluated	4 (3.4%)	0 (0.0%)	
Synchronous neoplasia	16 (13.8%)	8 (11.6%)	0.667
Multiple metachronous neoplasia	20 (17.2%)	6 (8.7%)	0.106

Note: Data are presented as n (%), mean ± SD, or median [IQR], as appropriate. IQR, interquartile range; WD, well differentiated; MD, moderately differentiated; PD, poorly differentiated; SRC, signet-ring cell carcinoma; *H. pylori*, *Helicobacter pylori*.

**Table 3:** Univariate and Multivariate Regression Analysis for Late Metachronous Neoplasia.

Variable	Univariate Analysis			Multivariate Analysis		
	OR	95% CI	*p*-Value	OR	95% CI	*p*-Value
Age (per 1 year)	0.955	0.920–0.992	0.018	0.967	0.929–1.007	0.101
Serum albumin (per 1 g/dL)	3.866	1.135–13.173	0.031	1.824	0.343–9.708	0.481
PNI (per 1 unit)	1.081	1.015–1.152	0.016	1.040	0.953–1.135	0.377
Undifferentiated type	12.984	1.562–107.950	0.018	10.727	1.274–90.352	0.029

Note: Continuous predictors were retained as continuous variables. OR, odds ratio; CI, confidence interval; PNI, prognostic nutritional index.

**Table 4:** Baseline Characteristics According to Timing of Metachronous Cancer.

Variable	Early (n = 59)	Late (n = 43)	*p*-Value
Age (years)	67.6 ± 8.0	64.9 ± 8.1	0.097
Gender			0.829
Male	49 (83.1%)	35 (81.4%)	
Female	10 (16.9%)	8 (18.6%)	
Family history of GC	7 (11.9%)	6 (14.0%)	0.755
Smoking			0.723
Never smoker	35 (59.3%)	24 (55.8%)	
Past or current smoker	24 (40.7%)	19 (44.2%)	
Hypertension	29 (49.2%)	18 (41.9%)	0.466
Diabetes mellitus	19 (32.2%)	9 (20.9%)	0.208
Heart disease	6 (10.2%)	5 (11.6%)	1.000
Cerebrovascular accident	3 (5.1%)	1 (2.3%)	0.636
End-stage renal disease	0 (0.0%)	0 (0.0%)	1.000
Aspirin	6 (10.2%)	3 (7.0%)	0.730
Other antithrombotic agent	11 (18.6%)	5 (11.6%)	0.336
Height (m)	1.6 ± 0.1	1.6 ± 0.1	0.862
Weight (kg)	64.7 ± 9.4	67.5 ± 8.5	0.110
BMI (kg/m^2^)	23.9 ± 2.6	24.9 ± 2.9	0.058
WBC count (/µL)	6600 [5550–7700]	6900 [6100–8850]	0.125
Neutrophil count (/µL)	3682.5 [3238–4459.5]	4121 [3293.5–4987]	0.410
Lymphocyte count (/µL)	2040.8 [1615.3–2437.6]	2233 [1583.1–2810.8]	0.257
Platelet count (×10^3^/µL)	208.8 ± 59.3	216.5 ± 54.8	0.501
NLR	1.88 [1.36–2.41]	1.76 [1.35–2.59]	0.989
PLR	98.76 [79.74–124.89]	94.99 [74.59–123.03]	0.616
ESR (mm/hr)	12 [5.2–22.8]	13 [7.5–22]	0.520
Albumin (g/dL)	4.20 ± 0.28	4.27 ± 0.22	0.156
PNI	52.4 ± 4.4	54.4 ± 6.0	0.069
Total cholesterol (mg/dL)	172.4 ± 34.9	169.8 ± 36.5	0.721
HDL (mg/dL)	50.9 ± 14.1	47.9 ± 13.7	0.309
LDL (mg/dL)	98.1 ± 31.0	97.0 ± 37.9	0.877
TG (mg/dL)	118 [83.5–154]	146.5 [84.2–212]	0.097
Fasting glucose (mg/dL)	104 [96–123]	107 [95–117]	0.909
TyG index	8.74 ± 0.55	8.93 ± 0.81	0.206
Serum creatinine (mg/dL)	0.83 [0.73–0.94]	0.81 [0.73–0.89]	0.444
CRP (mg/dL)	0.64 [0.38–1.97]	0.90 [0.40–1.96]	0.848

Note: Data are presented as n (%), mean ± SD, or median [IQR], as appropriate. SD, standard deviation; GC, gastric cancer; BMI, body mass index; WBC, white blood cell; IQR, interquartile range; NLR, neutrophil-to-lymphocyte ratio; PLR, platelet-to-lymphocyte ratio; ESR, erythrocyte sedimentation rate; PNI, prognostic nutritional index; HDL, high-density lipoprotein; LDL, low-density lipoprotein; TG, triglyceride; TyG, triglyceride–glucose index; CRP, C-reactive protein.

**Table 5:** Clinicopathologic Characteristics According to Timing of Metachronous Cancer.

Variable	Early (n = 59)	Late (n = 43)	*p*-Value
Lesion location			0.638
Lower third	33 (55.9%)	28 (65.1%)	
Mid third	23 (39.0%)	13 (30.2%)	
Upper third	3 (5.1%)	2 (4.7%)	
Lesion shape			0.760
Elevated (I, IIa)	14 (23.7%)	13 (30.2%)	
Flat (IIb)	22 (37.3%)	15 (34.9%)	
Depressed (IIc)	23 (39.0%)	15 (34.9%)	
Presence of ulcer	7 (11.9%)	4 (9.3%)	0.757
Tumor size (mm)	14 [9–22.5]	15 [10–24.5]	0.957
Atrophy			0.882
C1–2	1 (1.7%)	1 (2.3%)	
C3–O1	19 (32.2%)	12 (27.9%)	
O2–3	39 (66.1%)	30 (69.8%)	
Post-ESD diagnosis			0.320
WD adenocarcinoma	34 (57.6%)	21 (48.8%)	
MD adenocarcinoma	24 (40.7%)	18 (41.9%)	
PD adenocarcinoma	1 (1.7%)	3 (7.0%)	
SRC	0 (0.0%)	1 (2.3%)	
Differentiation			0.159
Differentiated type	58 (98.3%)	39 (90.7%)	
Undifferentiated type	1 (1.7%)	4 (9.3%)	
Lauren classification			0.336
Intestinal	58 (98.3%)	40 (93.0%)	
Diffuse	1 (1.7%)	2 (4.7%)	
Mixed	0 (0.0%)	1 (2.3%)	
Depth of invasion			1.000
Mucosa	55 (93.2%)	40 (93.0%)	
Submucosa	4 (6.8%)	3 (7.0%)	
Criteria			0.316
Absolute	32 (54.2%)	19 (44.2%)	
Expanded	27 (45.8%)	24 (55.8%)	
*H. pylori* infection			1.000
Negative or eradicated	51 (92.7%)	40 (93.0%)	
Persistent	4 (7.3%)	3 (7.0%)	
Unevaluated	4 (6.8%)	0 (0.0%)	
Synchronous neoplasia	10 (16.9%)	5 (11.6%)	0.454
Multiple metachronous cancer	9 (15.3%)	2 (4.7%)	0.113

Note: Data are presented as n (%), mean ± SD, or median [IQR], as appropriate. IQR, interquartile range; WD, well differentiated; MD, moderately differentiated; PD, poorly differentiated; SRC, signet-ring cell carcinoma; *H. pylori*, *Helicobacter pylori*.

**Table 6:** Univariate and Multivariate Logistic Regression Analysis for Late Metachronous Cancer.

Variable	Univariate Analysis			Multivariate Analysis		
	OR	95% CI	*p*-Value	OR	95% CI	*p*-Value
Age (per 1 year)	0.958	0.911–1.008	0.098	0.975	0.916–1.037	0.426
BMI (per 1 kg/m^2^)	1.160	0.996–1.352	0.057	1.110	0.931–1.324	0.245
WBC count (per 1000/µL)	1.234	0.993–1.534	0.058	1.165	0.885–1.534	0.276
PNI (per 1 unit)	1.081	0.996–1.172	0.063	0.990	0.883–1.109	0.859
Serum TG (per 10 mg/dL)	1.064	1.004–1.128	0.036	1.052	0.990–1.117	0.101
Undifferentiated type	5.949	0.641–55.246	0.117	4.682	0.416–52.679	0.211

Note: Continuous predictors were retained as continuous variables. WBC and TG odds ratios are expressed per 1000/µL and per 10 mg/dL increase, respectively. OR, odds ratio; CI, confidence interval; BMI, body mass index; WBC, white blood cell; PNI, prognostic nutritional index; TG, triglyceride.

**Table 7:** Baseline Characteristics According to Timing of Metachronous Adenoma.

Variable	Early (n = 56)	Late (n = 27)	*p*-Value
Age (years)	66.1 ± 7.9	62.3 ± 8.4	0.055
Gender			0.539
Male	40 (71.4%)	21 (77.8%)	
Female	16 (28.6%)	6 (22.2%)	
Family history of GC	5 (8.9%)	0 (0.0%)	0.168
Smoking			0.511
Never smoker	31 (55.4%)	17 (63.0%)	
Past or current smoker	25 (44.6%)	10 (37.0%)	
Hypertension	32 (57.1%)	13 (48.1%)	0.441
Diabetes mellitus	14 (25.0%)	7 (25.9%)	0.928
Heart disease	5 (8.9%)	2 (7.4%)	1.000
Cerebrovascular accident	4 (7.1%)	1 (3.7%)	1.000
End-stage renal disease	4 (7.1%)	0 (0.0%)	0.297
Aspirin	6 (10.7%)	4 (14.8%)	0.721
Other antithrombotic agent	8 (14.3%)	1 (3.7%)	0.259
Height (m)	1.6 ± 0.1	1.6 ± 0.1	0.582
Weight (kg)	66.2 ± 11.0	63.2 ± 10.7	0.251
BMI (kg/m^2^)	24.9 ± 3.7	24.1 ± 3.1	0.253
WBC count (/µL)	6000 [5375–7175]	6500 [5400–7400]	0.712
Neutrophil count (/µL)	3343 [2751.8–4382.8]	3719 [3166–4182]	0.333
Lymphocyte count (/µL)	2042.7 [1704.1–2319.8]	2077.5 [1597.3–2656.1]	0.478
Platelet count (×10^3^/µL)	230.9 ± 53.7	212.7 ± 52.5	0.149
NLR	1.69 [1.33–2.21]	1.81 [1.24–2.18]	0.911
PLR	115.12 [86.60–136.06]	96.49 [72.09–129.66]	0.123
ESR (mm/hr)	13 [7–20.8]	11 [6.5–19]	0.477
Albumin (g/dL)	4.14 ± 0.26	4.23 ± 0.23	0.117
PNI	51.7 ± 4.4	53.3 ± 5.0	0.161
Total cholesterol (mg/dL)	169.6 ± 36.6	180.5 ± 37.6	0.224
HDL (mg/dL)	46.6 ± 10.8	48.9 ± 14.2	0.471
LDL (mg/dL)	98.8 ± 35.0	102.2 ± 33.6	0.682
TG (mg/dL)	139.5 [97–178.5]	137 [94–185]	0.972
Fasting glucose (mg/dL)	107 [96.5–138.5]	100 [92.5–129.5]	0.225
TyG index	8.95 ± 0.62	8.85 ± 0.64	0.537
Serum creatinine (mg/dL)	0.82 [0.67–0.95]	0.78 [0.70–0.90]	0.502
CRP (mg/dL)	0.53 [0.27–1.43]	0.88 [0.55–1.63]	0.077

Note: Data are presented as n (%), mean ± SD, or median [IQR], as appropriate. SD, standard deviation; GC, gastric cancer; BMI, body mass index; WBC, white blood cell; IQR, interquartile range; NLR, neutrophil-to-lymphocyte ratio; PLR, platelet-to-lymphocyte ratio; ESR, erythrocyte sedimentation rate; PNI, prognostic nutritional index; HDL, high-density lipoprotein; LDL, low-density lipoprotein; TG, triglyceride; TyG, triglyceride–glucose index; CRP, C-reactive protein.

**Table 8:** Clinicopathologic Characteristics According to Timing of Metachronous Adenoma.

Variable	Early (n = 56)	Late (n = 27)	*p*-Value
Lesion location			0.916
Lower third	37 (66.1%)	19 (70.4%)	
Mid third	17 (30.4%)	7 (25.9%)	
Upper third	2 (3.6%)	1 (3.7%)	
Lesion shape			0.178
Elevated (I, IIa)	16 (28.6%)	9 (33.3%)	
Flat (IIb)	18 (32.1%)	13 (48.1%)	
Depressed (IIc)	21 (37.5%)	5 (18.5%)	
Presence of ulcer	6 (10.7%)	2 (7.4%)	1.000
Tumor size (mm)	16 [8–24]	16 [8–22]	0.861
Atrophy			0.960
C1–2	2 (3.6%)	1 (3.7%)	
C3–O1	19 (33.9%)	10 (37.0%)	
O2–3	35 (62.5%)	16 (59.3%)	
Post-ESD diagnosis			0.076
WD adenocarcinoma	33 (58.9%)	16 (59.3%)	
MD adenocarcinoma	23 (41.1%)	8 (29.6%)	
PD adenocarcinoma	0 (0.0%)	1 (3.7%)	
SRC	0 (0.0%)	2 (7.4%)	
Differentiation			0.032
Differentiated type	56 (100.0%)	24 (88.9%)	
Undifferentiated type	0 (0.0%)	3 (11.1%)	
Lauren classification			0.032
Intestinal	56 (100.0%)	24 (88.9%)	
Diffuse	0 (0.0%)	3 (11.1%)	
Mixed	0 (0.0%)	0 (0.0%)	
Depth of invasion			1.000
Mucosa	53 (94.6%)	26 (96.3%)	
Submucosa	3 (5.4%)	1 (3.7%)	
Criteria			0.803
Absolute	24 (43.6%)	11 (40.7%)	
Expanded	31 (56.4%)	16 (59.3%)	
*H. pylori* infection			0.463
Negative or eradicated	51 (91.1%)	23 (85.2%)	
Persistent	5 (8.9%)	4 (14.8%)	
Synchronous neoplasia	6 (10.7%)	3 (11.1%)	1.000
Multiple metachronous adenoma	5 (8.9%)	3 (11.1%)	0.711

Note: Data are presented as n (%), mean ± SD, or median [IQR], as appropriate. IQR, interquartile range; WD, well differentiated; MD, moderately differentiated; PD, poorly differentiated; SRC, signet-ring cell carcinoma; *H. pylori*, *Helicobacter pylori*.

**Table 9:** Univariate and Multivariate Logistic Regression Analysis for Late Metachronous Adenoma.

Variable	Univariate Analysis			Multivariate Analysis		
	OR	95% CI	*p*-Value	OR	95% CI	*p*-Value
Age (per 1 year)	0.943	0.888–1.000	0.052	0.937	0.881–0.997	0.040
CRP (per 1 mg/dL)	1.345	0.948–1.908	0.096	1.370	0.974–1.928	0.071
Undifferentiated type	Not estimable			Excluded		

Note: Undifferentiated type was not estimable because no undifferentiated-type cases were present in the early metachronous adenoma group and was therefore excluded from the multivariable model. Continuous predictors were retained as continuous variables. OR, odds ratio; CI, confidence interval; CRP, C-reactive protein.

## Data Availability

The data are not publicly available owing to institutional restrictions.

## Supplementary Materials

The following supporting information can be downloaded at https://www.mdpi.com/article/10.3390/jcm15218435/s1. Table S1: Sensitivity analysis using Firth penalized logistic regression for factors associated with late metachronous gastric neoplasia. Table S2: Sensitivity analysis using alternative cutoffs of 48 and 72 months for late metachronous gastric neoplasia: Firth penalized logistic regression. Table S3: Full versus reduced Firth logistic regression models in the cancer subgroup. Table S4: Bootstrap internal validation of the reduced cancer subgroup model.

## Abbreviations

The following abbreviations are used in this manuscript:

ESD	Endoscopic submucosal dissection
EGC	Early gastric cancer
*H. pylori*	*Helicobacter pylori*
NLR	Neutrophil-to-lymphocyte ratio
PLR	Platelet-to-lymphocyte ratio
PNI	Prognostic nutritional index
TyG	Triglyceride–glucose
OR	Odds ratio
CI	Confidence interval
