# Supplementary material for: Long-Term Surveillance After Curative Endoscopic Submucosal Dissection for Early Gastric Cancer: Distinct Clinicopathologic Characteristics of Early and Late Metachronous Neoplasia
Source: J Clin Med. 2026 Oct 29;15(21):8435. doi: 10.3390/jcm15218435 (PMC13764096; doi:10.3390/jcm15218435)
Supplement: Supplementary file 1 [file jcm-15-08435-s001.zip › jcm-4624376-supplementary.pdf]

Supplementary Table S1. Sensitivity analysis using Firth penalized logistic regression for factors associated with late metachronous gastric neoplasia.

Subgroup	Variable	Adjusted OR	95% CI lower	95% CI upper	*p*-value
**Overall**	Age	0.968	0.930	1.007	0.102
	Albumin	1.766	0.347	9.374	0.495
	PNI	1.039	0.954	1.133	0.381
	Undifferentiated histology	7.511	1.569	72.765	0.010
**Cancer**	Age	0.976	0.918	1.036	0.420
	BMI	1.102	0.933	1.311	0.252
	WBC per 1000	1.148	0.885	1.500	0.295
	PNI	0.990	0.888	1.105	0.847
	TG per 10	1.043	0.993	1.112	0.097
	Undifferentiated histology	3.479	0.495	39.912	0.214
**Adenoma**	Age	0.947	0.888	1.005	0.072
	CRP	1.340	0.996	1.934	0.053
	Undifferentiated histology	12.704	1.123	1749.115	0.039

OR, odds ratio; CI, confidence interval; PNI, prognostic nutritional index; BMI, body mass index; TG, triglyceride; CRP, C-reactive protein

Supplementary Table S2. Sensitivity analysis using alternative cutoffs of 48 and 72 months for late metachronous gastric neoplasia: Firth penalized logistic regression.

Cutoff (months)	Predictor	Early n	Late n	Adjusted OR	95% CI lower	95% CI upper	*p*-value
**48**	Age (per year)	97	87	0.986	0.949	1.023	0.450
	Albumin (per g/dL)	97	87	1.521	0.327	7.256	0.593
	PNI (per unit)	97	87	1.023	0.943	1.111	0.582
	Undifferentiated histology	97	87	5.247	1.106	50.609	0.036
**60**	Age (per year)	115	69	0.968	0.930	1.007	0.1024
	Albumin (per g/dL)	115	69	1.766	0.347	9.374	0.495
	PNI (per unit)	115	69	1.039	0.954	1.133	0.381
	Undifferentiated histology	115	69	7.511	1.569	72.765	0.010
**72**	Age (per year)	129	55	0.958	0.918	0.999	0.0423
	Albumin (per g/dL)	129	55	2.635	0.479	15.404	0.267
	PNI (per unit)	129	55	0.999	0.912	1.092	0.988
	Undifferentiated histology	129	55	3.184	0.805	14.289	0.098

OR, odds ratio; CI, confidence interval; PNI, prognostic nutritional index
Footnote: The numbers of patients in the early and late groups represent the entire study population (n = 185), whereas Firth penalized logistic regression was performed using complete cases (n = 184) because of one missing PNI value.

Supplementary Table S3. Full versus reduced Firth logistic regression models in the cancer subgroup

Subgroup	Variable	Adjusted OR	95% CI lower	95% CI upper	*p*-value
**Full 6-variables**	Age	0.976	0.919	1.036	0.420
	BMI	1.102	0.933	1.311	0.252
	WBC per 1000	1.148	0.885	1.500	0.295
	PNI	0.990	0.888	1.105	0.847
	TG per 10	1.043	0.993	1.112	0.097
	Undifferentiated histology	3.479	0.495	39.912	0.214
**Reduced 3-variables**	Age	0.968	0.917	1.020	0.226
	TG	1.052	1.002	1.120	0.039
	Undifferentiated histology	3.334	0.506	36.121	0.213

OR, odds ratio; CI, confidence interval; PNI, prognostic nutritional index; BMI, body mass index; TG, triglyceride; CRP, C-reactive protein

Supplementary Table S4. Bootstrap internal validation of the reduced cancer subgroup model.

Metric	Value	Interpretation
Apparent AUC	0.646	In-sample discrimination
Mean bootstrap optimism (AUC)	0.032	Bootstrap training minus original-sample test
Optimism-corrected AUC	0.614	Apparent minus mean optimism
Apparent log loss	0.632	In-sample log loss

AUC, area under curve

Footnote: Internal validation was performed using 500 bootstrap resamples of the reduced three-variable Firth logistic regression model. Model discrimination was assessed using the area under the receiver operating characteristic curve (AUC), and optimism-corrected AUC was calculated to account for potential overfitting. Of the 500 bootstrap resamples, 490 were evaluable for coefficient estimation because 10 resamples contained no patients with undifferentiated histology.
